# Occupational health literacy, injury risk, and temporary work disability: a multicenter cross-sectional study in Turkey

**DOI:** 10.1186/s12889-026-27277-5

**Published:** 2026-04-07

**Authors:** Yakup Celik, Gülden Sarı, Nilufer Merve Celik, Vahide Özban, Sinan Cem Uzunget, Ceprail Şimşek

**Affiliations:** 1 University of Health Sciences, Ankara Atatürk Sanatorium Training and Research Hospital, Ankara, Turkey; 2 Çubuk District Health Directorate, Ankara Provincial Health Directorate, Ankara, Turkey; 3 Ankara, Turkey

**Keywords:** Occupational health literacy, Occupational injuries, Metal industry, Occupational safety, Work disability, Return to Work, Multicenter studies

## Abstract

**Background:**

Occupational injuries continue to impose a substantial burden on workers’ health and productivity, particularly in the metal industry. However, the extent to which occupational health literacy (OHL) influences injury occurrence and work disability duration remains unclear.

**Methods:**

We conducted a multicenter cross-sectional study involving 649 workers from foundry and machinery factories in Turkey in April 2025. Two outcomes were assessed: risk of occupational injury and duration of temporary work disability. Time to first injury among blue-collar men hired after 2020 was analyzed using Cox regression. Lost workdays among all blue-collar men were modeled using negative binomial mixed models, applying a staged modeling approach: Model 1 included demographic and work-related factors, and Model 2 additionally included injury-related variables.

**Results:**

During the study period, 205 workers experienced 326 injury events. Among blue-collar men hired after 2020, the one- and three-year injury-free survival rates were 78.4% and 57.4%, respectively. Older age, higher educational attainment, and being single were associated with lower injury risk, whereas foundry work was associated with a higher risk of occupational injury. OHL was not associated with injury risk. In Model 1 of the negative binomial mixed models, higher OHL scores were associated with fewer lost workdays (IRR = 0.98; 95% CI: 0.97–1.00; *p* = 0.042); this association was attenuated and no longer statistically significant after adjustment for injury-related characteristics in Model 2.

**Conclusions:**

Higher OHL was not associated with injury occurrence but may be associated with the duration of temporary work disability following injury. These findings suggest that work disability duration reflects a multifactorial process shaped by both injury-specific characteristics and individual-level factors, with OHL potentially playing a complementary role in post-injury recovery rather than serving as a primary determinant of outcomes. Longitudinal studies are needed to clarify these relationships and inform targeted workplace health interventions.

**Supplementary Information:**

The online version contains supplementary material available at 10.1186/s12889-026-27277-5.

## Introduction

Occupational injuries remain a major global public health concern, affecting both individual well-being and economic productivity [1]. Beyond fatal cases, non-fatal injuries contribute substantially to the global burden through temporary work disability and lost workdays. According to the International Labour Organization (ILO), occupational accidents and diseases account for nearly 4% of the world’s gross domestic product (GDP), primarily due to productivity losses from temporary labor incapacity. However, the role of workers’ occupational health literacy (OHL) in these outcomes has not been clearly defined [2, 3].

The metal industry, characterized by high physical, ergonomic, and environmental hazards, ranks among the sectors with the highest injury rates globally and in Turkey [4–6]. A meta-analysis of 15 studies reported a combined prevalence of occupational injuries in the iron and steel industries of approximately 55%, indicating that nearly one in two workers experiences an occupational injury in this sector [7]. Data from Turkey’s Social Security Institution (2012–2022) reveal a similar pattern: the primary metal and machinery manufacturing sectors are among the top five industries with the highest number of injuries. In the primary metal industry, 8.9 of every 100 workers sustained an occupational injury, ranking it second after mining in terms of both incidence and lost workdays. Although machinery manufacturing ranked just below the primary metal industry in injury severity, it accounted for a large number of lost workdays due to the overall high injury frequency [6, 8]. Despite this well-documented burden, little is known about how workers’ cognitive and behavioral capacities, particularly occupational health literacy, are associated with these outcomes. Prior research has linked general health literacy to improved self-care and preventive behaviors; however, the specific role of OHL in relation to injury occurrence and recovery in high-risk industrial sectors remains unclear.

Health literacy (HL) refers to an individual’s ability and motivation to access, understand, evaluate, and apply health information to maintain and promote health [9]. Occupational health literacy (OHL) is a specific dimension of HL that enables workers to recognize hazards, follow safe work practices, and use occupational health information to make informed safety decisions [10, 11]. Unlike general HL, which broadly applies to personal health management, OHL emphasizes hazard recognition, safe work practices, and navigation of workplace health systems within increasingly complex industrial environments [10, 12]. OHL is considered to play a role in shaping workers’ decision-making processes, risk perception, and safety-related behaviors [10]. Lower levels of OHL have been associated with misinterpretation of safety instructions, improper use of personal protective equipment, and continuation of unsafe work practices [11].

Temporary work disability refers to the total number of calendar days a worker is unable to perform work within a year, excluding the day of the accident. Depending on the national reporting system, these days are termed “days away from work” (Occupational Safety and Health Administration) or “days lost” (European Statistics on Accidents at Work). Although terminology varies, the measure consistently reflects the degree of functional labor loss caused by occupational injury [13, 14].

While several studies have examined factors affecting occupational injuries in the metal industry [7], health literacy among industrial workers [15, 16], and workplace health promotion training programs [17], research addressing both health literacy and occupational injuries simultaneously remains limited [18, 19]. To our knowledge, no previous study has examined the association between OHL and both the occurrence and consequences of occupational injuries among workers in the primary metal and machinery manufacturing sectors.

Therefore, this multicenter cross-sectional study examined whether higher OHL among metal industry workers is associated with a lower risk of occupational injury and a shorter duration of temporary work disability.

## Methods

### Study design and setting

We conducted a multicenter cross-sectional study in four factories—two foundries and two machinery manufacturing plants—located across three industrial sites in Turkey in April 2025. This study examined the association between occupational health literacy (OHL) and both the risk of occupational injury and the duration of temporary work disability. Including factories from different sites enhanced the heterogeneity of production processes and improved sample representativeness. In this study, “site” refers to a geographically distinct industrial area hosting one or more factories. In total, 725, 230, and 156 employees worked at Sites 1, 2, and 3, respectively, totaling 1,111 workers across all sites.

### Study population and sample

We calculated the required sample size (*n* = 545) using Cochran’s finite population formula in OpenEpi (version 3.01) with a 95% confidence interval (CI), a 3% margin of error, a prevalence estimate of *p* = 0.5, and a design effect of 1.0 [20]. We selected a conservative prevalence estimate of 50% to maximize the required sample size in the absence of prior population-specific estimates of occupational injury prevalence. We applied stratified sampling by site because factories at the same site shared an occupational health and safety unit, reporting system, training protocols, and personnel lists, and accident records were more easily accessible at the site level.

Using proportional stratified sampling, we planned to recruit 356 participants from Site 1, 113 from Site 2, and 76 from Site 3. Accounting for a potential 15% data loss, the target sample size was set at 642 participants. Participation was voluntary; 670 employees agreed and completed the questionnaire. After excluding participants with substantial missing data (e.g., incomplete sociodemographic information or OHL scale responses), 649 participants remained for analysis (413 from Site 1, 137 from Site 2, and 99 from Site 3). The distribution of participants across sites remained similar to that of the original population after data cleaning.

We treated factory type (foundry or machinery) as a covariate in all multivariate models, independent of the site, to control for any differences that might have resulted from stratification. Permanent employees aged ≥ 18 years were included, whereas subcontracted workers were excluded because their accident records were unavailable.

### Data collection process

We piloted the questionnaire with 57 employees before data collection and revised it based on feedback to produce the final version. The survey assessed participants’ sociodemographic and work-related characteristics and OHL levels. Accident histories from January 1, 2020, to April 30, 2025, were obtained from official factory occupational health unit reports, following the Social Security Institution (SGK) “SGK-032 Work Accident and Occupational Disease Report Form” [21].

Trained researchers conducted paper-based, face-to-face interviews in April 2025. Participation was voluntary. Each participant received a unique identification code to maintain confidentiality; no personal identifiers were recorded. The survey took approximately 10 min to complete.

### Measurements / variables

#### Dependent variables

Two dependent variables were analyzed in this study. The first was whether the participant had experienced an occupational injury, which was used as the dependent variable in the survival analyses. The second was the number of days with temporary work-related disability, calculated as “the total number of calendar days lost due to temporary incapacity for work, excluding the day of the accident,” according to the definitions of the SGK and the ILO [3, 6].

#### Independent variables

The independent variables were obtained from two complementary sources: (i) a self-administered questionnaire completed by the participants and (ii) workplace administrative records, including personnel files and official SGK-032 occupational injury reports. All non-subjective questionnaire items (age, marital status, education, job position, factory, department, work tenure, and injury history) were cross-checked against institutional records to ensure accuracy.

Sociodemographic variables included age (years), marital status (married/single), educational level (primary school, middle/secondary school, high school, or higher education), and household income level (income less than/equal to/greater than expenses). Household income was based on self-reports, whereas the remaining sociodemographic variables were verified using personnel files.

Work-related variables included job position (blue-collar/white-collar), factory type (foundry or machinery), department, shift schedule (yes/no), overtime frequency (always/often/sometimes/rarely/never), and total work experience. Work experience was calculated as the time elapsed between the employment start date and either the date of injury (for injury-related analyses) or the date of data collection (for baseline descriptive analyses). Shift schedules and overtime were obtained from the questionnaire, whereas job position, factory type, department, and work tenure were confirmed using administrative records.

To characterize injury events, accident type and injured body region were coded according to the Social Security Institution’s Statistical Yearbook (2024) [6], based on information extracted from the SGK-032 forms. The number of temporary work disability days was obtained exclusively from official SGK records. For analysis, injury-related variables were categorized before regression analyses. Injured body part was grouped into four categories: upper extremity, lower extremity, head/eye, and trunk or multiple body regions, based on anatomical proximity and clinical similarity to ensure sufficient observations per category. Accident nature was categorized into five groups: cut/crush/entrapment, struck/collision/falling object, falls, exposure/overexertion, and other rare mechanisms. Low-frequency categories (e.g., electrical injuries, explosions) were combined to improve model stability and avoid sparse data bias in multivariable analyses.

Occupational health literacy (OHL) was included as an independent variable in all multivariable models. OHL was assessed using the total score of the Occupational Health Literacy Scale (OHLS), originally developed by Suthakorn et al. and adapted into Turkish by Uskun et al. The scale comprises 38 items across four subdimensions, each rated on a 3-point Likert scale (1 = least appropriate; 3 = most appropriate), with a total score range of 38–114. Higher scores indicate better OHL. The OHLS does not define validated cut-off values; therefore, OHL was analyzed as a continuous variable to reflect the original scale structure and retain full data variability. The Turkish version demonstrated excellent internal consistency (Cronbach’s α = 0.93) [10, 22]. Due to copyright restrictions, OHLS items were not included in the supplementary file; only questionnaire items used in analyses were provided.

### Statistical analyses

We performed all statistical analyses using SPSS 26.0 (IBM Corp., Armonk, NY, USA) and R software (R Foundation for Statistical Computing, Vienna, Austria, version 4.5.1).

Continuous variables were summarized as mean ± standard deviation and median (minimum–maximum), and categorical variables were summarized as numbers and percentages. Participants were categorized into four groups based on sex and occupational classification: blue-collar women, blue-collar men, white-collar women, and white-collar men. As the vast majority of occupational injuries occurred among blue-collar men, subsequent analyses were restricted to this subgroup.

We estimated the time to the first occupational injury among blue-collar men who started working after 2020 using Kaplan–Meier analysis and calculated 1-year and 3-year accident-free survival rates. We compared the survival times between groups using the log-rank test. To identify predictors of occupational injury, we fitted Cox proportional hazards regression models with age, marital status, educational level, income, factory type, shift work, and the total OHL score as independent variables. We determined the model variables based on bivariate analyses and previous literature and used the backward likelihood ratio method.

We fitted generalized linear mixed models (GLMMs) to identify factors associated with the number of temporary work disability days among all blue-collar men between January 2020 and May 2025, including those who were employed prior to 2020. We first tested Poisson models but found them unsuitable because of excessive dispersion in the dependent variable. Therefore, we used a negative binomial distribution (nbinom2 parameterization). To account for multiple accident records belonging to the same worker, we included the individual identification number as a random intercept. The random effect significantly improved the model fit.

We applied a staged modeling strategy. In Model 1, we included sociodemographic variables (age, marital status, education, and income) and workplace variables (factory type, shift work, overtime, and work experience), along with the OHL score. In Model 2, we additionally included variables for injured body region and type of accident to examine whether the association between OHL and lost workdays persisted after adjustment for injury characteristics.

We evaluated model assumptions using simulation-based residual diagnostics with the DHARMa package. We detected no residual overdispersion in the fitted models. Residual diagnostics indicated adequate model fit. Although we observed slight deviation in zero values in the Model 2, zero-inflated negative binomial models did not improve model fit and were not retained. We conducted all GLMM analyses using the glmmTMB package in R (v4.3.1) [23]. Additional analyses did not suggest meaningful non-linearity in the association between OHL and lost workdays.

We present the findings using Kaplan–Meier survival curves, hazard ratios (HR, 95% CI) from Cox regression, and incidence rate ratios (IRR, 95% CI) from GLMMs. We fitted all models using complete-case analysis and did not perform imputation for missing data. We set statistical significance at *p* < 0.05.

## Results

Table 1 summarizes the characteristics of the study population (*n* = 649). The mean age of the participants was 38.0 ± 8.7 years; 95.5% were men, 71.6% were married, and 56.6% were high school graduates. Most participants were blue-collar workers (88.6%), and 78.4% worked in shifts. The median occupational health literacy (OHL) score was 80.5 (range: 38–114). Because blue-collar men constitute the majority, subsequent analyses focused on this group.

**Table 1:** Sociodemographic and occupational characteristics of participants and occupational health literacy scores, (*N* = 649)

Variable	*N* (%)	Mean ± SD / Median (min- max)
Sex		
Male	620 (95.5)	
Female	29 (4.5)	
Marital status		
Married	465 (71.6)	
Single	184 (28.4)	
Education level		
Elementary school	48 (7.4)	
Middle school/secondary	136 (21.0)	
High school	368 (56.6)	
Higher education	97 (14.9)	
Income status		
Income less than expenses	319 (49.2)	
Income equal to expenses	258 (39.8)	
Income greater than expenses	72 (11.1)	
Occupational classification		
Blue-collar	575 (88.6)	
White-collar	74 (11.4)	
Factory type		
Foundry	343 (52.9)	
Machinery	242 (37.3)	
Other (administrative/support staff and unclassified workers.)	64 (9.9)	
Shift work		
Shift work	509 (78.4)	
Non-shift	140 (21.6)	
Age		38.00 ± 8.74 / 38.05(19.87–65.04)
Occupational Health Literacy score		83.36 ± 15.56 / 80.5(38–114)

*Abbreviations*: *SD* Standard deviation, *min* minimum, *max* maximum

Between January 1, 2020, and April 30, 2025, 326 occupational injuries were documented. Injuries were more frequent in foundry factories (68.1%), particularly in the core-making (40.5%) and cleaning (34.7%) departments. In machinery factories, the milling section accounted for most of the injuries (75.8%). The most common injury types were being struck by a moving object (24.8%), falling or overturning an object (21.5%), and being caught or crushed by an object (21.5%). Injuries mainly involved the upper extremities (43.6% of cases). The mean duration of temporary work disability was 17 days, with a median of 6 days. The detailed injury characteristics are presented in Table 2. The mean age of the injured workers was 34.3 ± 7.2 years old.

**Table 2:** Characteristics of occupational injuries and distribution of injury-related factors, (*N* = 326)

	*N* (%)	Mean ± SD / Median (min- max)
Factory where the injury occurred,	326	
Foundry factory	222 (68.1)	
Machinery factory	99 (30.4)	
Unclassified^a^	5 (1.5)	
Departments in foundry factory,	222	
Core making	90 (40.5)	
Cleaning	77 (34.7)	
Molding	13 (5.9)	
Quality control	13 (5.9)	
Pattern workshop	11 (5.0)	
Melting	10 (4.5)	
Others^b^	8 (3.5)	
Departments in machinery factory,	99	
Milling	75 (75.8)	
Quality control	10 (10.1)	
Washing	6 (6.1)	
Others^b^	8 (8.0)	
Events leading to injury,	326	
Struck by moving object / collision	81 (24.8)	
Falling or overturning object	70 (21.5)	
Caught in / crushed by	70 (21.5)	
Fall on the same level	26 (8.0)	
Overexertion / excessive physical load	24 (7.4)	
Exposure to hazardous substances	18 (5.5)	
Cut / pierced by sharp object	15 (4.6)	
Struck against stationary object	13 (4.0)	
Fall from height	2 (0.6)	
Fire or explosion	2 (0.6)	
Electrical injury	1 (0.3)	
Unspecified or other injuries^c^	4 (1.2)	
Body part injured,	326	
Upper extremity	142 (43.6)	
Lower extremity	88 (27.0)	
Head	67 (26.5)	
Back / lumbar	15 (4.6)	
Multiple regions	6 (1.8)	
Trunk	5 (1.5)	
Unspecified	3 (0.9)	
Temporary work disability days		16.67 ± 24.02 / 6 (0-139)

*Abbreviations*: *SD* Standard deviation, *min* minimum, *max* maximum

^a^“Unclassified” refers to safety and cleaning staff performing shared duties for both the foundry and machinery facilities at Location 1

^b^“Other” includes low-frequency or hard-to-classify departments (e.g., maintenance, warehouse)

^c^“Unspecified or other injuries” includes type of event not clearly recorded

During the study period, 205 of the 649 employees experienced 326 injuries. Among them, 139 had one injury, 39 had two injuries, and 27 had three or more injuries. Overall, 319 of the 326 injuries (97.9%) occurred among blue-collar men. At the individual level, 198 of the 569 blue-collar men (34.8%) experienced at least one occupational injury. Among workers hired after 2020 (*n* = 245), 202 were blue-collar men, of whom 82 (40.6%) experienced at least one injury.

Using Kaplan–Meier analysis, we examined time to first occupational injury among blue-collar men hired after 2020. The mean injury-free survival time was 34.5 ± 1.3 months (95% CI: 31.8–37.1). Injury-free survival rates were 78.4% at one year and 57.4% at three years. No statistically significant differences were observed by marital status, education, income, factory type, or shift work (all *p* > 0.05). Detailed survival probabilities and log-rank test results by subgroup are shown in Table 3.

**Table 3:** Factors influencing the time to first occupational injury among blue-collar male workers hired after 2020: Kaplan–Meier analysis and log-rank test results

	*N* Injured / Total	Mean injury-free survival (months) ± SE	95% CI	1-year survival (%)	3-year survival (%)	Log-Rank *p*
Total	82 / 202	34.48 ± 1.34	31.83 − 37.13	78.4	57.4	
Marital status *0.99*						
Single	37 / 97	34.27 ± 2.03	30.29–38.24	76.8	55.5	
Married	45 / 105	34.69 ± 1.82	31.11–38.26	80.0	58.9	
Education Level						*0.41*
Elementary school	6 / 9	25.44 ± 5.98	13.73 − 37.16	66.7	33.3	
Middle school/secondary	28 / 58	32.97 ± 2.45	28.17–37.77	74.1	57.1	
High school	40 / 112	35.45 ± 1.84	31.85 − 39.05	81.0	56.5	
Higher education	8 / 23	36.28 ± 3.81	28.79 − 43.76	81.7	70.7	
Income status						*0.29*
Income less than expenses	53 / 117	32.63 ± 1.74	29.21–36.04	77.6	54.7	
Income equal to expenses	20 / 64	37.60 ± 2.34	33.02–42.19	85.7	66.2	
Income greater than expenses	9 / 21	33.78 ± 4.11	25.73 − 41.83	81.0	49.6	
Factory Type						*0.17*
Foundry	58 / 126	33.36 ± 1.70	30.03–36.69	77.6	55.2	
Machinery	24 / 76	36.20 ± 2.16	31.97 − 40.43	79.8	62.1	
Shift work						*0.17*
Shift work	80 / 191	34.04 ± 1.40	31.30–36.78	77.7	56.6	
Non-shift work	2 / 11	39.73 ± 4.18	31.54 − 47.91	-	-	
Overtime						*0.98*
Always	4 / 10	33.33 ± 6.13	21.33–45.34	90.0	58.3	
Often	10 / 28	36.35 ± 3.43	29.62 − 43.07	92.6	42.1	
Sometimes	45 / 108	33.36 ± 1.81	29.82 − 36.91	95.4	52.9	
Rarely	17 / 44	34.92 ± 3.06	28.93 − 40.92	90.9	61.5	
Never	6 / 12	34.03 ± 4.20	25.81 − 42.26	91.7	31.3	

*Abbreviations*: *SE* Standard error, *CI* Confidence interval

The multivariable Cox regression model was used to identify independent predictors of injury-free survival. The hazard of occupational injury decreased by approximately 12% for each additional year of age (HR, 0.88; 95% CI: 0.83–0.92; *p* < 0.001). Single workers had a 52% lower risk of injury than married workers (HR, 0.48; 95% CI: 0.27–0.86; *p* = 0.01). Higher educational levels were also associated with lower injury risk; the hazard ratios among high school and higher-education graduates ranged from 0.25 to 0.30 (all *p* < 0.05). By factory type, foundry workers faced an approximately 1.8-fold higher hazard than machinery workers (HR, 1.80; 95% CI: 1.05–3.12; *p* = 0.03). Income status, shift work, and OHL scores were not significantly associated with injury-free survival. Multivariable Cox regression results are summarized in Table 4.

**Table 4:** Multivariable Cox regression analysis of factors influencing the time to first occupational injury among 202 blue-collar male workers hired after 2020

	*N* (Injured / Total)	HR^a^	95% CI	*p*
Age	82 / 202	0.88	0.83–0.92	*< 0.001*
Marital Status				
Married (ref)	45 / 105	1		*–*
Single	37 / 97	0.48	0.27–0.86	*0.01*
Education Level				
Elementary school (ref)	6 / 9	1		*–*
Middle school / secondary	28 / 58	0.30	0.09–0.93	*0.04*
High school	40 / 112	0.25	0.10–0.63	*0.003*
Higher education	8 / 23	0.28	0.11–0.73	*0.01*
Factory Type				
Machinery (ref)	58 / 126	1		*–*
Foundry	24 / 76	1.80	1.05–3.12	*0.03*

*Abbreviations*: *HR* Hazard ratio, *CI* Confidence interval

^a^Occupational health literacy, shift work, and income level were included as additional variables in the model

Table 5 presents results from negative binomial generalized linear mixed models examining factors associated with the number of lost workdays following occupational injuries among blue-collar male workers. In Model 1, which included sociodemographic and workplace variables, higher OHL scores were associated with fewer lost workdays. Each one-point increase in the OHL score reduced the expected number of lost workdays by approximately 2% (IRR 0.98, 95% CI 0.97–1.00, *p* = 0.042). None of the remaining covariates showed a statistically significant association with lost workdays. In Model 2, after adjusting for injury-related variables, including injured body part and accident type, the association between OHL and lost workdays was attenuated and no longer statistically significant (IRR 0.99, 95% CI 0.97–1.00, *p* = 0.127). Furthermore, other sociodemographic and workplace variables remained non-significant. Injury-related factors were significantly associated with lost workdays. Compared with head/eye injuries, upper extremity injuries (IRR 9.54, 95% CI 5.04–18.06), lower extremity injuries (IRR 8.17, 95% CI 4.38–15.21), and trunk or multiple body region injuries (IRR 3.62, 95% CI 1.59–8.27) were associated with longer work disability durations. Regarding accident type, struck/collision/falling object injuries were associated with longer lost workdays compared with cut/crush/entrapment injuries (IRR 1.79, 95% CI 1.05–3.03); other categories were not statistically significant.

**Table 5:** Factors affecting the number of lost workdays following occupational injuries among blue-collar male workers: results of negative binomial generalized linear mixed models (GLMMs)

Variable	Model 1^a^			Model 2^b^		
	*N*	IRR (95% Cl)	*p*	*N*	IRR (95% Cl)	*p*
Age		1.03 (0.98–1.08)	*0.19*		1.04 (1.00–1.09)	*0.06*
Occupational health literacy scale score		0.98 (0.97–1.00)	*0.04*		0.99 (0.97–1.00)	*0.13*
Marital status						
Married	237	1	*–*	233	1	*–*
Single	82	0.99 (0.52–1.84)	*0.94*	80	1.04 (0.57–1.87)	*0.91*
Income status						
Income less than expenses	200	1	*–*	198	1	*–*
Income equal to expenses	87	0.59 (0.35–1.04)	*0.06*	84	0.64 (0.39–1.07)	*0.09*
Income greater than expenses	32	1.52 (0.72–3.22)	*0.27*	31	1.38 (0.70–2.73)	*0.36*
Education level						
Elementary school	23	1	*–*	23	1	*–*
Middle school/secondary	97	1.09 (0.44–2.74)	*0.85*	97	0.82 (0.35–1.92)	*0.65*
High school	175	0.62 (0.26–1.51)	*0.30*	169	0.53 (0.23–1.20)	*0.13*
Higher education	24	1.09 (0.34–3.54)	*0.87*	24	0.83 (0.28–2.43)	*0.73*
Factory type						
Machinery	99	1	*–*	94	1	*–*
Foundry	220	1.46 (0.86–2.49)	*0.16*	219	1.53 (0.93–2.51)	*0.09*
Shift work						
Shift work	283	1	*–*	278	1	*–*
Non-shift work	36	0.61 (0.27–1.35)	*0.22*	35	0.88 (0.41–1.88)	*0.75*
Overtime						
Always	19	1	*–*	19	1	*–*
Often	35	1.31 (0.44–3.94)	*0.63*	35	1.04 (0.38–2.85)	*0.94*
Sometimes	173	0.64 (0.25–1.68)	*0.37*	169	0.55 (0.23–1.34)	*0.19*
Rarely	66	0.75 (0.26–2.15)	*0.59*	64	0.55 (0.21–1.45)	*0.22*
Never	26	0.71 (0.21–2.41)	*0.59*	26	0.66 (0.21–2.04)	*0.47*
Work experience (months)		1.00 (1.00–1.01)	*0.20*		1.00 (1.00–1.01)	*0.23*
Injured body part						
Head/eye				63	1	*–*
Upper extremity				141	9.54 (5.04–18.06)	*< 0.001*
Lower extremity				84	8.17 (4.38–15.21)	*< 0.001*
Trunk/multiple body regions				25	3.62 (1.59–8.27)	*0.002*
Accident nature						
Cut/crush/entrapment				82	1	*–*
Struck/collision/falling object				159	1.79 (1.05–3.03)	*0.03*
Falls				27	1.81 (0.81–4.05)	*0.15*
Exposure/overexertion				39	1.34 (0.63–2.86)	*0.45*
Other rare mechanisms				6	1.46 (0.32–6.72)	*0.62*

Six observations excluded in Model 2 due to missing injury-related data

*Abbreviations*: *IRR* Incidence rate ratio, *CI* Confidence interval

^a^Model 1 includes age, occupational health literacy score, marital status, income status, education level, factory type, shift work, overtime, and work experience (months)

^b^Model 2 additionally includes injured body part (grouped) and accident nature (grouped)

## Discussion

This multicenter cross-sectional study examined the association between occupational health literacy (OHL) and both the risk of occupational injury and the duration of temporary disability following accidents among workers in Turkey’s primary metal and machinery manufacturing sector. Although OHL was not associated with injury risk, our findings suggest that it may be related to post-injury outcomes. According to the staged modeling approach, in Model 1, which included individual sociodemographic and workplace organizational factors (factory type, shift work, overtime, and work experience), higher OHL scores were associated with shorter durations of lost workdays. Each one-point increase in the OHL score was associated with an approximately 2% lower number of lost workdays in Model 1. Previous studies on return-to-work outcomes suggest that individual capacities such as education, self-efficacy, and recovery expectations have been associated with more favorable post-injury trajectories [24, 25]. A study conducted in Turkey found that workers who did not lose workdays following occupational injury had higher health literacy levels, consistent with our findings [19]. Several mechanisms may help explain this observed association. Cancelliere et al. (2016) identified individual factors such as self-efficacy, recovery expectations, educational attainment, and socioeconomic status as important determinants of return-to-work outcomes [24]. Although OHL was not examined in this study, the individual factors identified reflect broader individual-level characteristics that, similar to OHL, may influence how workers manage their health and recovery processes. In addition, coordinated communication between healthcare providers and workplaces has been highlighted as a key component of successful return-to-work processes [24]. Nutbeam’s classification of health literacy into functional, interactive, and critical domains emphasizes that progression from functional to interactive and critical levels requires not only basic information acquisition, but also more advanced cognitive and social skills, including interpretation of information, decision-making, and social interaction [26]. In this context, higher OHL may be associated with greater social and communication capacities, which could, in turn, be linked to earlier return-to-work or shorter duration of temporary work disability. Consistent with the return-to-work literature, individual factors and coordinated workplace–healthcare interactions appear to play a key role in shaping recovery trajectories. However, given the cross-sectional design and the timing of OHL measurement after injury, these interpretations should be interpreted cautiously. Most prior research has focused on return-to-work outcomes [24, 25]. A distinctive aspect of this study is the evaluation of total temporary disability duration after occupational injury, defined by the ILO as the total number of calendar days lost in a year due to an accident, excluding the day of the accident, representing the magnitude of accident-related labor-force losses [3]. From a broader perspective, improving OHL may complement efforts to reduce individual and societal economic losses associated with occupational injuries, alongside interventions targeting injury severity. In Model 2, which additionally adjusted for injury-related characteristics, the association between OHL and lost workdays was attenuated and no longer statistically significant, although the direction of the association remained consistent. This finding is consistent with the strong and proximal prognostic role of injury-related characteristics in determining the duration of work disability, and is in line with evidence from systematic reviews on return-to-work outcomes showing that injury severity and functional impact are among the most important predictors of recovery trajectories [24, 25]. In this context, Model 2 was specified to assess the association between OHL and work disability duration under comparable injury conditions defined by injured body part and accident nature. In nonlinear count models such as negative binomial regression, estimates obtained after including additional covariates may reflect a more conditional relationship, whereas models with more limited adjustment may reflect a broader association [27]. Therefore, differences between models with varying levels of covariate adjustment may reflect differences in the interpretation of the estimated association rather than a true change in the relationship itself. Taken together, it is plausible that work disability duration reflects a multifactorial process shaped by both injury-specific characteristics and individual capacities at different levels.

In contrast to its association with post-injury outcomes, occupational health literacy (OHL) was not associated with the occurrence of occupational injuries. This finding aligns with mixed evidence from previous studies. For instance, Rauscher and Myers (2014) reported a positive association between OHL and injury prevalence among adolescent workers, attributing this to greater exposure to training in hazardous jobs or differences in training quality [18]. Another explanation is that individuals with higher OHL may recall or report injuries more accurately. Importantly, the timing of OHL measurement should be considered when interpreting these results. In this study, OHL was assessed after injuries occurred, limiting the ability to determine the direction of the association. On one hand, higher OHL may promote safer behaviors and reduce injury risk. On the other, workplace accidents may increase workers’ awareness and knowledge, leading to higher OHL levels. Therefore, the absence of an observed association should be interpreted cautiously, as it may reflect temporal ambiguity and potential reverse causation rather than a true lack of relationship between OHL and injury prevention.

Our study also confirmed several previously established associations. Consistent with the multifactorial nature of injury risk, older age and higher education levels were associated with lower injury risk, consistent with meta-analyses indicating that experience and awareness improve safety performance [28, 29]. The higher injury risk among foundry workers aligns with previous studies documenting elevated physical workload, dust exposure, and complex production processes in foundries [5, 30, 31].

The lower risk of occupational injury observed among single workers in this study has been infrequently reported and warrants further investigation [32–34]. Most prior studies have reported either a protective effect of marriage for certain outcomes, such as return to work, or inconsistent findings [32]. For example, Santos et al. reported higher return-to-work rates among married workers; however, sensitivity analyses showed this association lost significance when specific studies were excluded, indicating substantial heterogeneity [32]. This discrepancy may reflect differences in exposure patterns and occupational characteristics across worker groups [34]. In addition, psychosocial factors, including work–family interactions, have been suggested as potentially relevant in previous research; however, these were not directly measured in the present study [34]. Therefore, the observed association should be interpreted cautiously, and its underlying mechanisms remain unclear.

The generalizability of our results was strengthened by the multicenter design and inclusion of diverse production environments in the metal and machinery industries. Assessing both injury incidence and temporary disability days using the same dataset allowed us to explore two interconnected dimensions that are rarely examined together in the occupational health literature. Furthermore, the use of official workplace records minimized recall bias, an important methodological advantage. To our knowledge, this study may be the first to quantitatively examine the association between occupational health literacy (OHL) and post-injury work disability duration.

Despite its strengths, this study had several limitations. First, the temporal mismatch between OHL measurement and injury occurrence limits the ability to determine the direction of the association and raises the possibility of reverse causation. Second, the cross-sectional assessment of OHL also constrains causal inference. Third, analyses focused primarily on blue-collar men, limiting generalizability to women and white-collar workers. Additionally, unmeasured variables such as injury severity, workplace safety culture, personality traits, and psychosocial factors could have influenced results. Finally, employees who left their jobs after injury were not included, potentially underrepresenting long-term disability cases and underestimating effect sizes.

The findings have several implications for occupational health practice but should be interpreted in light of the temporal mismatch between OHL measurement and injury occurrence. The higher injury risk observed in foundry settings further highlights the importance of addressing sector-specific hazards. Within this broader context, OHL may support risk awareness, hazard recognition, and adherence to safety procedures, but should be considered as one component within a wider system of organizational and environmental risk controls. Workplace interventions incorporating OHL-sensitive approaches—such as clear risk communication, tailored educational materials, and worker-centered safety instructions—may strengthen prevention and recovery efforts when integrated with structural safety measures. Overall, improving OHL may complement interventions targeting injury severity by supporting post-injury recovery and enhancing occupational health outcomes. In addition, it may contribute to reducing individual and societal economic losses associated with occupational injuries and may support broader goals related to economic sustainability and workforce productivity.

Future research using longitudinal designs should clarify the causal effects of OHL on occupational injuries and post-injury incapacity. Including more diverse sectors, female workers, and different marital status groups would improve the generalizability of the findings. Testing whether marital status influences outcomes through economic burden, family responsibilities, social support, or psychosocial stressors will further refine our understanding of these relationships.

In conclusion, this multicenter study suggests that OHL may be related to post-injury recovery processes; however, this association was attenuated after accounting for injury-related characteristics. Temporary work disability duration appears to be largely influenced by injury-specific factors, with OHL potentially playing a complementary role. No significant association was observed between OHL and injury occurrence. Younger age, lower educational attainment, and employment in foundry settings were associated with higher injury risk, indicating that injury occurrence in high-risk industrial settings is influenced by multiple factors at individual and workplace levels. Integrating OHL-sensitive approaches into workplace health strategies—such as clear risk communication and tailored educational materials—may support post-injury recovery when combined with broader organizational and environmental interventions. Overall, occupational injuries and their consequences appear influenced by a combination of structural, organizational, and individual factors, with OHL serving as a supportive component within a broader framework.

## Supplementary Information

Supplementary Material 1.

## Data Availability

The datasets generated and analyzed during the current study are not publicly available due to institutional data-protection policies but are available from the corresponding author on reasonable request.

## Abbreviations

OHL: Occupational Health Literacy
HL: Health Literacy
OHS: Occupational Health and Safety
ILO: International Labour Organization
OSHA: Occupational Safety and Health Administration
SGK: Social Security Institution (Sosyal Güvenlik Kurumu, Türkiye)
GLMM: Generalized Linear Mixed Model
IRR: Incidence Rate Ratio
HR: Hazard Ratio
CI: Confidence Interval
GDP: Gross Domestic Product
SPSS: Statistical Package for the Social Sciences
SD: Standard Deviation
LRT: Likelihood Ratio Test
DF: Degrees of Freedom
ESAW: European Statistics on Accidents at Work
