# Supplementary material for: Occupational health literacy, injury risk, and temporary work disability: a multicenter cross-sectional study in Turkey
Source: BMC Public Health. 2026 Apr 7;26:1607. doi: 10.1186/s12889-026-27277-5 (PMC13191891; doi:10.1186/s12889-026-27277-5)
Supplement: Supplementary file 1 — Supplementary Material 1. [file 12889_2026_27277_MOESM1_ESM.docx]

Supplementary File 1. Questionnaire Items Used in the Study (English Version)

Note:This supplementary file includes only the questionnaire items used in the study analyses. Items belonging to the copyrighted Occupational Health Literacy Scale (Turkish version) are not reproduced here, in accordance with copyright restrictions. Participant identifiers, consent text, and administrative instructions were removed in accordance with journal policy.

Section A. Sociodemographic Information

1. What is your age?

2. What is your marital status?

1. Married

2. Single

3. What is your highest level of education?

1.Elementary school

2. Middle school/secondary

3. High school

4. Higher education

4. How would you evaluate your monthly household income level?

1. My income is less than my expenses

2. My income is equal to my expenses

3. My income is greater than my expenses

Section B. Employment Information

5. Which of the following best describes your current job?

1. Blue-collar (production/shop-floor workers)

2. White-collar (office/technical/administrative worker)

3. Other (please specify): _________

6. In which factory do you currently work?

1.Foundry

2.Machinery

3.Other (please specify): __________

7. In which department do you currently work? __________

8. How long have you been working in this workplace? _____ years / _____ months

Section C. Work Schedule and Overtime

9. Do you work in shifts?

1. Yes

2. No

10. How often do you work overtime?

1. Always

2. Often

3. Sometimes

4. Rarely

5. Never

Section D. Occupational Injury History

11. Have you ever experienced an occupational injury at this workplace?

1. Yes

2. No

Note:Self-reported injury history was cross-checked with official SGK-032 injury records during data validation.
